# Supplementary material for: From non-use to covert and overt use of contraception: Identifying community and individual factors informing Nigerian women’s degree of contraceptive empowerment
Source: PLoS One. 2020 Nov 18;15(11):e0242345. doi: 10.1371/journal.pone.0242345 (PMC7673533; doi:10.1371/journal.pone.0242345)
Supplement: Attachment — Submitted filename: Response to Reviewers.docx [file pone.0242345.s001.docx]

**PONE-D-20-19653
From non-use to covert and overt use of contraception: identifying community and individual factors informing Nigerian women’s degree of contraceptive empowerment**

Responses to Reviewers’ Comments

| **S/N** | **Reviewer #1 Comments** | **Response** |
| --- | --- | --- |
| 1. | “My primary comment is about the use of the word empowerment in relation to covert use. Various results presented in the study compare non-use with covert use - this can show a somewhat skewed perspective on the empowerment of covert use. Covert use remains a reflection of disempowerment of women, in any society. I feel that this could be phrased more appropriately and the dangerous of covert use should be explicitly stated. Covert use is a threat to continued use of contraception, and therefore is a risk factor for unintended pregnancies. While it is true that covert users may be more empowered than non-users, this should be phrased with caution.” | We appreciate this perspective and have added information on the threats associated with covert use in lines 150-155 to address this important nuance. |
| 2. | “The conclusion in line 315-318 is not well supported by the data. While it is clear that male partners will not be informed of the covert use, it is not clear that the overt users in the community are aware of covert users using contraception. Covert users may well feel inferior or scared to reveal their contraceptive use to overt users, if the overt users occupy a more powerful position in society (as the authors claim). These covert users tended to be single compared to overt users who are married or in relationships. This power dynamic can have a significant influence on younger, single women overtly using contraception.” | Thanks for sharing this perspective. We have edited the statement to read, “Additionally, women who reside in communities where more women used FP report more covert than non-use, possibly as a result of an enabling environment that normalizes use and facilitates access to FP.” Please see lines 331-333. |
| 3. | “Please check the titles of the tables - some where a little misleading, especially table 3 and 4 where the comparative results were not included. There are also four columns of data in each table that are not clearly labelled.” | Thank you for this observation. Table and column titles have been edited accordingly. |
| 4. | “Lines 137-140 will benefit from a reference.” | A reference has been inserted. The updated lines are 151-155. |

| **S/N** | **Reviewer #2 Comments** | **Response** |
| --- | --- | --- |
| 1. | “It could be useful to provide a brief definition of covert use at the start of the background prior to describing why women may have chosen to use contraception in this way.” | A definition has been added on lines 71-72: “Covert use, the use of a contraceptive method without the knowledge of a woman’s partner, may represent…” |
| 2. | “In the background, it is briefly mentioned that reproductive coercion, partner violence, and misperception of partners’ attitudes are reasons for covert use. If available, it may be useful for the authors to provide some information on the reasons women choose to use covertly in this specific cultural context, as this may vary depending on the setting.” | Motivations for covert use in this specific context have been added, on lines 110-114. |
| 3. | “In lines 184 – 187, the way authors have defined whether participants had need for contraception could be clarified. As it is written, it is currently unclear whether those that had not recently used contraception and were sexually active were considered to have need regardless of desire to become pregnant or only if they were not trying to become pregnant.” | A statement on lines 202 to 204 has been added to clarify: “Women who desired a child within the 2-year period following the survey were not considered to be in need for contraception and were excluded from the analytic sample.” |
| 4. | “The authors mention in the discussion that age and parity are factors that generally grant women status and as a results, increases their empowerment. It may be interesting to comment on why in this study age was not found to relate to covert use vs no use or covert use vs overt use and why parity was not found to associate with covert vs overt use.” | A comment has been added in lines 339-342: “Even though our results do not reach statistical significance to show an association between older age (35-49 years) and more covert versus non-use, or covert versus overt use, or an association between high parity and more covert versus overt use, the effect estimates are in the expected positive direction.” |
| 5. | “While this study certainly lends important detail to the literature on covert use, because fertility goals and contraceptive use preferences/intentions may be dynamic it may be important to take care not to refer to nonuse, covert use, and overt use as a continuum as in lines 397-401. This may be particularly true given that data used were cross-sectional.” | We appreciate this perspective and have changed the word “continuum” to “spectrum” throughout the paper. We agree that the data we have may not be able to truly reflect a continuum, given its cross sectional nature. |
